# Osteoarticular infection caused by *Talaromyces Marneffei* and *Salmonella* in a person living with HIV: a case report

**DOI:** 10.1186/s12879-023-08554-9

**Published:** 2023-08-28

**Authors:** Lele Yu, Binhai Zhang, Jinchuan Shi, Mengyan Wang, Hu Wan

**Affiliations:** Department II of Infectious Diseases, Affiliated Hangzhou Xixi Hospital, Zhejiang University School of Medicine, Hangzhou, China

**Keywords:** *Talaromyces marneffei*, *Salmonella*, HIV, Case report, NGS

## Abstract

**Introduction:**

*Talaromycosis* is a common invasive fungal disease in patients with HIV. However, its association with bone destruction is unusual in AIDS patients with talaromycosis.

**Case Presentation:**

This report covers the case of a 38-year-old male AIDS patient coinfected with *Talaromyces marneffei* and *Salmonella*. The case, which involved bone destruction, was identified via metagenomic next-generation sequencing (mNGS). Following treatment with a combination of amphotericin B and piperacillin-tazobactam, the patient’s elbow motion noticeably improved. Imaging findings revealed that the progression of bony destruction had halted.

**Conclusion:**

Bone damage due to *Talaromyces marneffei* infection is infrequent in HIV-positive patients. Therefore, healthcare professionals must be vigilant for potential bone lesions associated with this type of infection. Prompt diagnosis and antimicrobial treatment are crucial.

## Introduction

*Talaromyces marneffei* (*T*. *marneffei)*, previously known as *Penicillium marneffei*, is an opportunistic pathogen common in Southeast Asian regions including China, Thailand, Vietnam, Laos, Myanmar, and India [1]. The pathogen can cause *talaromycosis* (TSM), a potentially fatal disease characterized by an insidious onset and complex, variable symptoms. It can infect the skin, respiratory system, digestive system, and reticuloendothelial system, leading to localized or disseminated infection. First reported in Thailand in 1984, bone destruction due to *T*. *marneffei* is extremely rare [2, 3]. Co-infection of bone and joints have never been reported.

In this report, we present a case of *T*. *marneffei* infection impacting the bone and joint of an AIDS patient. We also review the clinical characteristics to enhance clinical understanding.

## Case presentation

A 38-year-old man was admitted to our hospital, presenting with a 2-month history of rash and half a day of abnormal behavior. This behavior was characterized by the patient standing at his door wearing only underwear, talking to himself, and appearing confused. He also had a history of unsafe sexual practices.

**Fig. 1:**
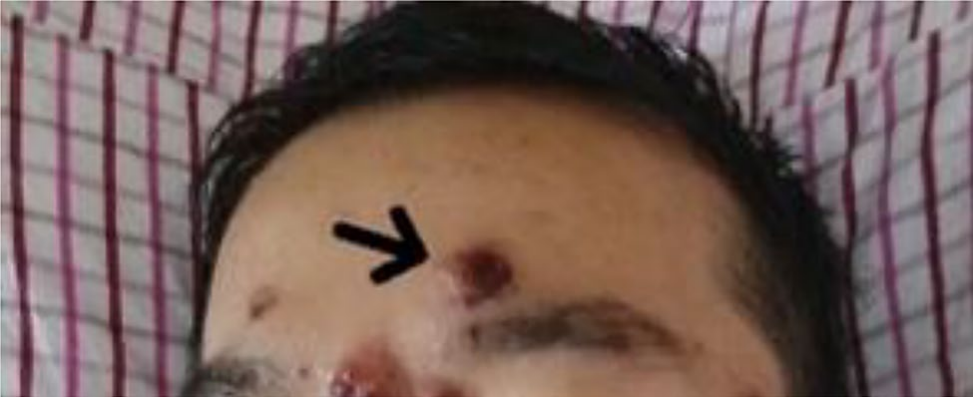
The patient’s face exhibits numerous necrotic papules

Physical examination yielded the following findings: a temperature of 38.9 °C, pulse rate of 144 beats/min, respiratory rate of 40 breaths/min, and blood pressure of 90/55 mmHg. The patient exhibited signs of delirium, with unfocused gaze, irrelevant responses, and neck resistance. Palpable superficial lymph nodes were found in the neck, armpits, and groin, each measuring approximately 1.5*1 cm. These nodes were irregularly arranged, soft, and had a good range of motion. A substantial number of necrotic papules were visible on the patient’s face and trunk (Fig. 1).

Laboratory examinations revealed low levels of hemoglobin (97 g/L), leukocytes (2.5 × 10^9^/L), and platelets (49 × 10^9^/L). High levels were observed for C-reactive protein (159.27 mg/L), lactic acid (7.04 mmol/L). Anti-HIV antibody was positive, and the patient’s CD4 T-cell count was 6 cells/ml. The patient’s HIV RNA level was 8.76 × 10^4^ IU/ml.

Additional blood tests, including those for cytomegalovirus-DNA, Toxoplasma-IgM, and cryptococcal capsular polysaccharide antigen, were within the normal range. Parvovirus B19-IgG/IgM and rapid plasma reagin (RPR) tests were negative.

Given the patient’s abnormal behavior, and no significant abnormalities observed in the head CT scan, we performed a lumbar puncture examination. The cerebrospinal fluid (CSF) was pale yellow, with significantly elevated protein levels (2369 mg/L) and cell count (nucleated cells: 600 × 10^4^/L, 90% neutrophils), but decreased glucose (1.1 mmol/L) and chloride (112 mmol/L) levels. All other CSF tests, including IgG, IgM, IgA and ADA were within the normal range. Tests for CMV-DNA, ink stain of CSF, Cryptococcus capsular polysaccharide antigen, and acid-fast bacilli returned negative results.

Chest computed tomography (CT) showed multiple enlarged lymph nodes (abdominal, retroperitoneal, bilateral axillary, and supraclavicular). Blood cultures tested positive for *Talaromyces marneffei* (identified after 6 days of culture) and *Salmonella* (identified after 3 days of culture). Concurrently, CSF culture suggested a *Salmonella* infection. The culture results indicated a multidrug-resistant *Salmonella* Group D, resistant to ampicillin and sulbactam, gentamicin, amikacin, cefazolin, cefotetan, and tobramycin, but sensitive to piperacillin-tazobactam, ceftazidime, ciprofloxacin, ceftriaxone, cefepime, imipenem, compound sulfamethoxazole, ertapenem and aztreonam.

The patient, in septic shock, was promptly administered aggressive anti-septic shock therapy with empirical treatment. Concurrently, he received antifungal therapy with intravenous voriconazole (200 mg twice daily) for 10 days, and intravenous meropenem (2 g every 12 h) for 10 days due to the severity of his condition, with meningitis concurrent with sepsis. This was followed by oral voriconazole (200 mg twice daily) and intravenous ceftriaxone (2 g every 12 h) for 23 days. Symptomatic treatment was administered continuously during hospitalization. With active treatment and basic care, the patient achieved complete remission of fever, delirium. A biopsy was not performed on the rash, which recovered gradually with treatment. After infection control was relatively stabilized (2 weeks after admission), antiviral therapy with tenofovir, lamivudine, and dolutegravir was initiated.

Unexpectedly, three weeks after treatment initiation, the patient’s left elbow started showing slight swelling and pain. At this time, anteroposterior and lateral X-rays of the left elbow joint showed regular bone arrangement, complete bone structure, normal shape, and no density difference. The joint was in position, and no obvious abnormality was detected in the soft tissue. Ultrasound revealed no significant abnormalities in the left upper limb’s axillary, brachial, basilic, and median cubital veins.

**Fig. 2:**
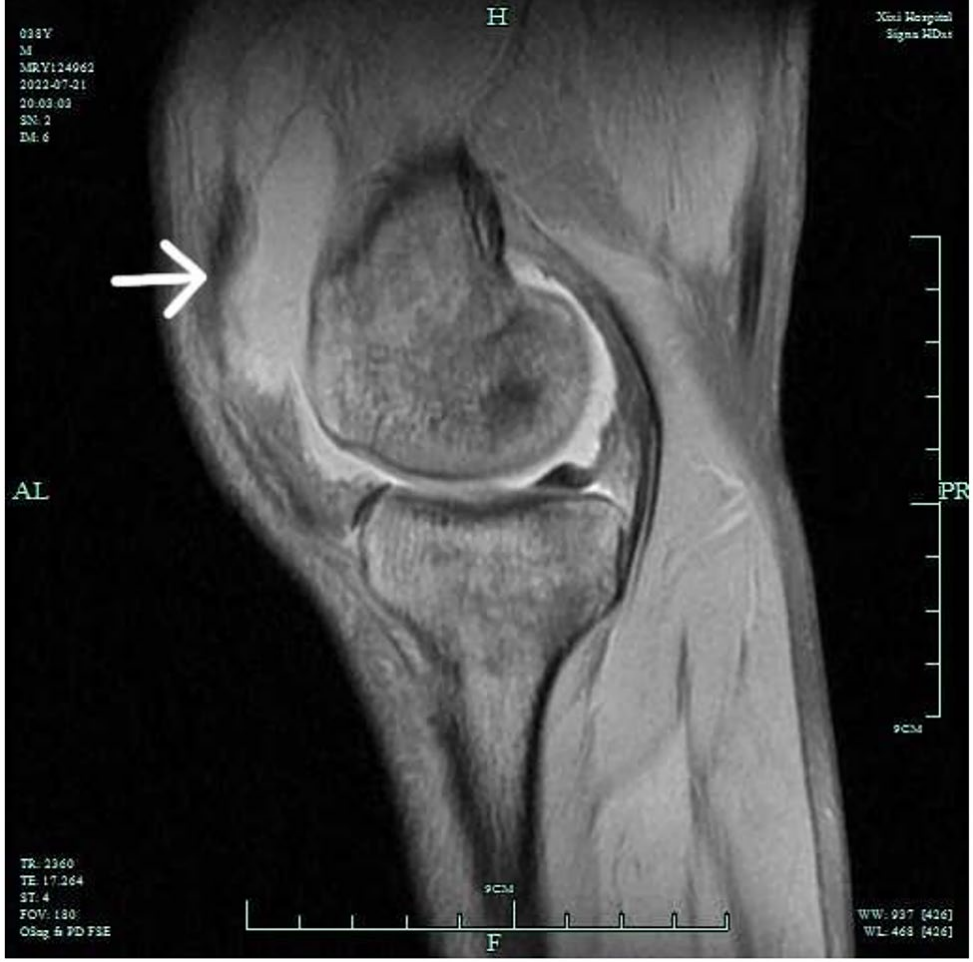
MRI of the left knee joint revealed numerous patchy long T1 and long T2 signal lesions in the knee bones, with indistinct edges. Abundant fluid signals were noted in the joint space and upper abdominal bursae, accompanied by swelling of the surrounding muscles and soft tissues

**Fig. 3:**
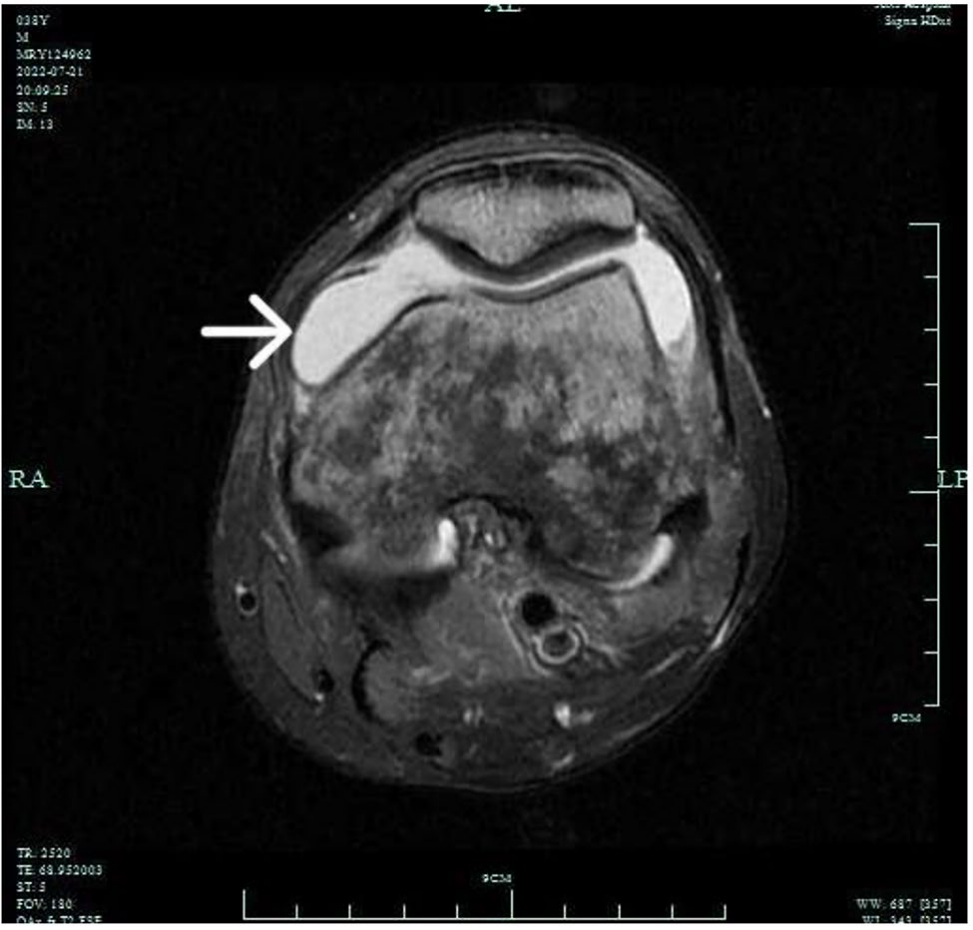
Transverse axis MRI of the left knee joint revealed numerous patchy long T1 and long T2 signal lesions in the knee bones, with indistinct edges. Abundant fluid signals were noted in the joint space and upper abdominal bursae, accompanied by swelling of the surrounding muscles and soft tissues

Over the following week, more joint symptoms manifested. The left knee and elbow joints became swollen, tender to touch, and exhibited limited range of motion in flexion and extension. Magnetic resonance imaging (MRI) of the left knee revealed numerous patchy long T1 and long T2 signal lesions in the knee bones, with unclear edges. A large amount of fluid signal was observed in the joint space and the upper bursae of the abdomen, and swelling was observed in the surrounding muscles and soft tissues (Figs. 2 and 3).

**Fig. 4:**
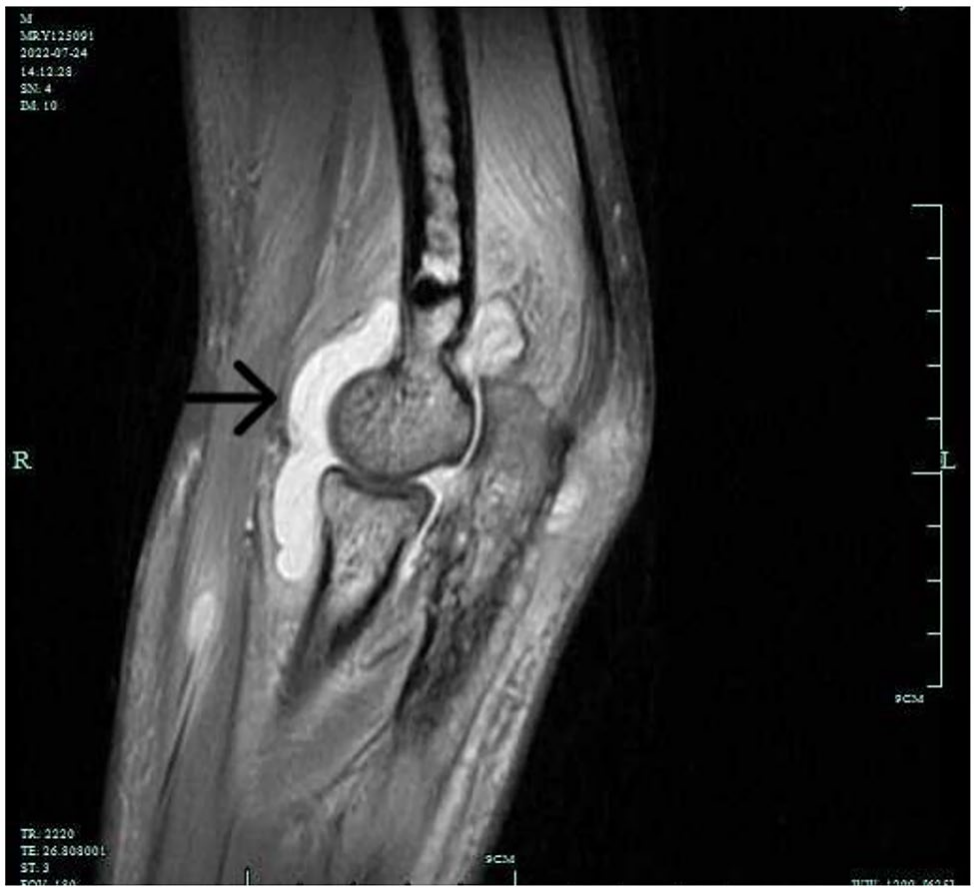
MRI of the left elbow joint displayed diffuse long T1 and long T2 signals in each group of left elbow osteoplasia, large slices of long T2 signals in the surrounding muscle tissue, blurred muscle space, spots of long T2 signals in the triceps tendon, and significant fluid accumulation in the left elbow joint space

**Fig. 5:**
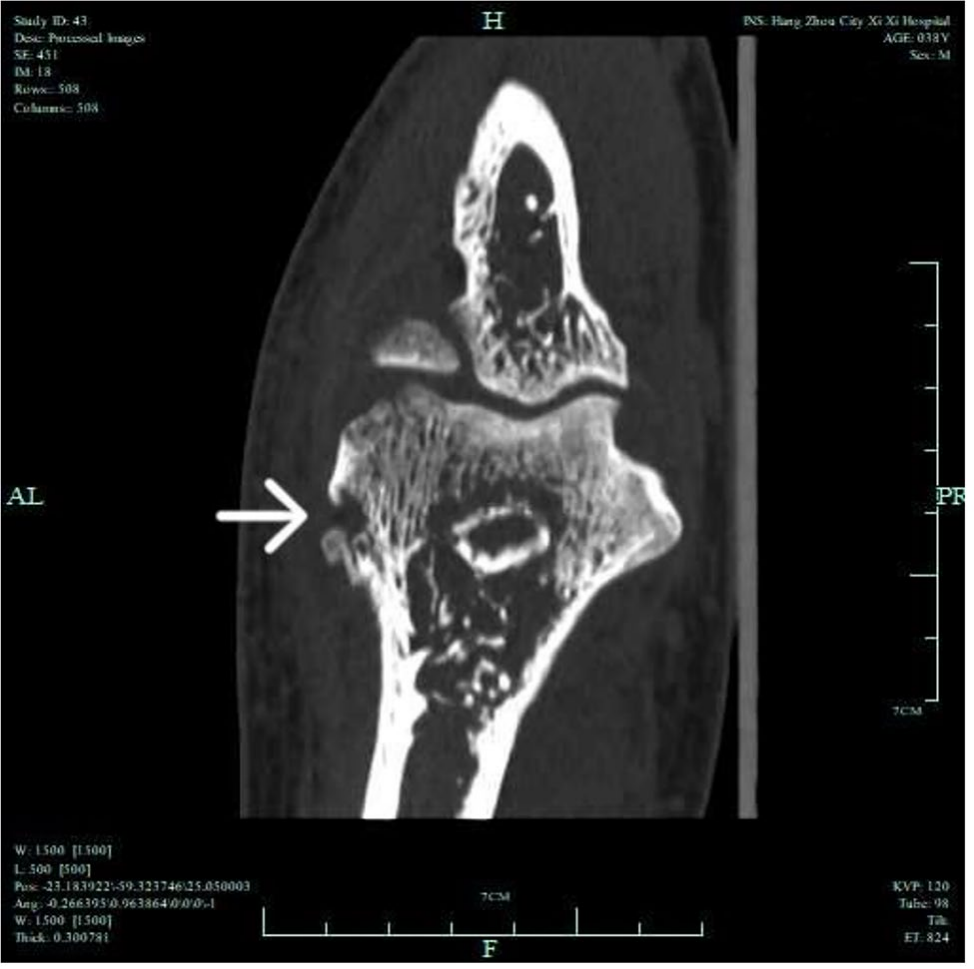
CT scan of the left elbow joint exhibited a reduced density area of worm erosion in the lower end of the humerus and the olecranon cortex of the ulna. The trabecular structure was disorganized, the left elbow was properly positioned, surrounding soft tissue was swollen, and fluid was visible in the joint space

Fluid accumulated in the joint cavity and suprapatellar bursa of the left knee. The MRI of the left elbow joint showed diffuse long T1 and long T2 signals in each group of left elbow osteoplasia. Large slices of long T2 signals were observed in the muscle tissue around the left elbow, with blurred muscle space, spots of long T2 signals in the triceps tendon, and a significant amount of fluid in the left elbow joint space (Fig. 4). The CT scan of the left elbow joint revealed a reduced density area of erosion in the lower end of the humerus and olecranon cortex of the ulna. The trabecular structure was disorganized, the left elbow was properly positioned, the surrounding soft tissue was swollen, and fluid was visible in the joint space (Fig. 5).

We sought the assistance of an orthopedist to conduct arthrocentesis of the elbow joint and knee. The results indicated an increased cell count (nucleated cell count was 7200 × 10^4^/L, neutrophils 79%) in the left knee joint. Similarly, the left elbow joint puncture fluid demonstrated elevated levels of cells (nucleated cell count was 96,044 × 10^4^/L, neutrophils 77%), aiding the diagnosis of osteoarthritis. The etiology inspection of the puncture fluid tests, including brucella, HLA-B27, autoantibody, vasculitis, acid-fast bacilli, fungi, and bacteria detection, didn’t provide significant information. Nevertheless, MRI of the joint revealed swelling and effusion in the left elbow joint space, and progressive bone destruction at the left lower end of the humerus and the left olecranon. During this period of joint-related symptoms, the patient exhibited normotension, fever (temperature repeatedly reached 38.8 °C), and HR 80–120. His respiration and blood oxygen saturation remained stable.

Under such conditions, we performed metagenomic next-generation sequencing (mNGS) to identify potential pathogens in the puncture fluid due to the disease’s progression. Given the lack of standard interpretation method for mNGS results and the reported parameter diversity across different sequencing platforms, we used GenSeizer™-based mNGS results for interpretation. The results revealed 8000 *T*. *marneffei* and 300 *Salmonella*-specific sequences. Consequently, we adjusted his final diagnosis to fungal arthritis caused by *T*. *marneffei* and *Salmonella*. It was considered that the anti-infection effect was not good in the early stage with ceftriaxone and voriconazole. We altered his treatment to an intravenous injection of amphotericin B (0.5 mg/(kg·d)for one month)) and piperacillin-tazobactam. Due to poor performance of ceftriaxone sodium in the preliminary results, we switched to piperacillin/tazobactam based on the resistance pattern. The patient’s swollen joint symptoms improved after one month of admission following extensive anti-infection treatment, and he returned to normal temperature. Upon discharge, he continued treatment with itraconazole and cefixime. At a two-month follow-up, we observed significant improvement in elbow movement. Imaging findings suggested no further progression of bone destruction. HIV RNA was below detectable levels, and the CD4 + T count increased to 22/µl.

## Discussion

*Talaromyces marneffei* is a severe, deeply pathogenic fungus. It exhibits a rare dimorphism with a hyphal state (propagating phase) at 25 to 28 °C and a yeast state (pathogenic phase) at 37 °C. The bamboo rat is the natural host of the fungu. *Talaromyces marneffei* is the most common invasive fungal disease in HIV-infected patients in Southeast Asian countries and southern China and is frequently seen in immunocompromised individuals. Upon entering the human body, *T*. *marneffei* is first engulfed by mononuclear macrophages, where it proliferates within these macrophages and disseminates through the reticuloendothelial system. Over the past 20 years, the number of immunodeficient hosts has increased due to widespread use of immunosuppressive agents, organ transplantation, AIDS, etc., leading to a rise in *Talaromyces marneffei* disease. Nonetheless, due to a lack of specific clinical features, the misdiagnosis rate remains high, often leading to various complications and a high mortality rate.

*Talaromyces marneffei* infection can be categorized as localized or disseminated. Localized infection often confines lesions to the lungs, skin, and lymph nodes—the initial invasion sites of the pathogen—with negative blood culture. Disseminated infection is highly lethal and presents with atypical early symptoms such as recurrent fever, cough, expectoration, emaciation, and anemia. As the infection progresses, the bacteria can disseminate via lymph or blood to various body organs, causing abscess-like lesions in the skin, brain, bone marrow, and internal organs.

The current standard treatment regimens for talaromycosis patients are amphotericin B (0.7–1.0 mg/kg/day for 2 weeks) and itraconazole (200 mg twice daily for 10 weeks). However, amphotericin B is highly nephrotoxic and can cause other side effects. Relevant literature reports no significant difference in mortality between the amphotericin B and itraconazole groups when initiating intravenous therapy in AIDS patients with *Talaromyces marneffei* [4]. Another study used voriconazole to treat seven cases of disseminated *Talaromycosis marneffei*, resulting in significant symptom relief. This study suggests that voriconazole is as effective as amphotericin B and itraconazole in treating *Talaromycosis marneffei* and that voriconazole is less toxic and safer in the treatment of talaromycosis. Therefore, voriconazole may also be a preferred treatment option for talaromycosis [5].

Talaromycosis combined with bone destruction primarily occurs in non-AIDS patients. A retrospective study from China included 100 patients diagnosed with disseminated PSM, consisting of 65 HIV-infected patients and 35 HIV-negative patients. Only 14 patients had osteolytic lesions, all of whom were HIV-negative [6]. Radiographs and CT scans displayed multiple radiations such as erosive bone destruction, periosteal hyperplasia, fractures, and swelling of the surrounding soft tissues. Bone pain is a characteristic symptom of patients with bone destruction. Young and middle-aged people with underlying diseases and a long disease course are at high risk [7]. We described what we believe is the first reported of osteoarticular injury caused by *Talaromyces marneffei* and *Salmonella* in an HIV-positive individual. The presentation, diagnosis, and treatment of osteoarticular injury did not significantly differ compared to co-infected non-HIV cases.

However, bone destruction in talaromycosis often occurs late in the disease course, necessitating early diagnosis. mNGS can simultaneously sequence a vast number of DNA molecules, yielding results within 24 to 72 h. It diagnoses by integrating and analyzing all nucleic acid sequences of isolates and hosts and determining the species and abundance of microorganisms in conjunction with clinical features. mNGS can successfully identify infectious pathogens of unknown origin in samples like blood, bone marrow, and gastrointestinal tract. Compared with culture-based methods, mNGS offers significant advantages in terms of high detection efficiency and speed. The detection accuracy and positive rate of microorganisms with mNGS are higher [8]. Liu [9] reports that the diagnostic sensitivity of mNGS is 100%, and the specificity is 98.7%.

Clinically, histopathology, staining, and cultures should be performed first and if these tests do not reveal any specific organisms, then mNGS should be considered as a further test to be performed. When *T. marneffei* infection is suspected, clinicians can attempt mNGS to confirm the diagnosis [10]. For talaromycosis patients with multiple systemic lesions, clinicians should consider conducting histopathology simultaneously or performing mNGS cultures at multiple sites. Clinicians should closely monitor disease progression to reduce the occurrence of clinical misdiagnosis or missed diagnosis.

In addition, in the report, the patient’s osteoarticular Infection occurred after HAART, it was therefore difficult to distinguish the immune reconstitution inflammatory syndrome (IRIS) or a refractory infection.

Bone damage due to *T. marneffei* infection is infrequent in HIV-positive patients. Therefore, healthcare professionals must be vigilant for potential bone lesions associated with this type of infection. Prompt diagnosis and antimicrobial treatment are crucial.

## Data Availability

The raw data supporting the conclusions of this article will be made available by the authors without undue reservation. For data inquiries, please contact yll8324@163.com.
